# Methoxy-Poly(ethylene glycol) Modified Poly(L-lactide) Enhanced Cell Affinity of Human Bone Marrow Stromal Cells by the Upregulation of 1-Cadherin and Delta-2-catenin

**DOI:** 10.1155/2014/738239

**Published:** 2014-04-14

**Authors:** Xueli Mao, Zetao Chen, Junqi Ling, Jingjing Quan, Hui Peng, Yin Xiao

**Affiliations:** ^1^Department of Operative Dentistry and Endodontics, Guanghua School of Stomatology, Hospital of Stomatology, Sun Yat-sen University, 56 Ling Yuan Road West, Guangzhou 510055, China; ^2^Guangdong Provincial Key Laboratory of Stomatology, 56 Ling Yuan Road West, Guangzhou 510055, China; ^3^Australia-China Centre for Tissue Engineering and Regenerative Medicine, Queensland University of Technology, Brisbane, 60 Musk Avenue, Kelvin Grove, Brisbane, QLD 4059, Australia; ^4^Institute of Health and Biomedical Innovation, Queensland University of Technology, Brisbane, 60 Musk Avenue, Kelvin Grove, Brisbane, QLD 4059, Australia; ^5^Australian Institute for Bioengineering and Nanotechnology, The University of Queensland, Corner College and Cooper Roads, Brisbane, QLD 4072, Australia

## Abstract

Poly(l-lactide) (PLLA), a versatile biodegradable polymer, is one of the most commonly-used materials for tissue engineering applications. To improve cell affinity for PLLA, poly(ethylene glycol) (PEG) was used to develop diblock copolymers. Human bone marrow stromal cells (hBMSCs) were cultured on MPEG-*b*-PLLA copolymer films to determine the effects of modification on the attachment and proliferation of hBMSC. The mRNA expression of 84 human extracellular matrix (ECM) and adhesion molecules was analyzed using RT-qPCR to understand the underlying mechanisms. It was found that MPEG-*b*-PLLA copolymer films significantly improved cell adhesion, extension, and proliferation. This was found to be related to the significant upregulation of two adhesion genes, CDH1 and CTNND2, which encode 1-cadherin and delta-2-catenin, respectively, two key components for the cadherin-catenin complex. In summary, MPEG-*b*-PLLA copolymer surfaces improved initial cell adhesion by stimulation of adhesion molecule gene expression.

## 1. Introduction

Tissue engineering is under development as a method for repairing and regenerating defective or damaged tissues and organs. A scaffold is a critical part of this approach, providing three-dimensional structures with biomimic microenvironments for the functional behaviors of stem cells which are subsequently delivered to the defective areas. Many materials have been investigated for the fabrication of scaffolds. Poly(l-lactide) (PLLA), a versatile biodegradable polymer, is one of the most commonly used materials for tissue engineering applications, because of its advantage of being eco-friendly and its excellent biocompatibility, processibility, and low energy dependence properties [1]. However, vital challenges remain to be overcome in order to make PLLA a desirable scaffold material, one of which is related to poor cell adhesion and spreading [2].

The attachment of mammalian cells onto scaffold materials is a key step for the implantation of cells and the formation of cell/matrix complexes. Adhesion to biomaterials is mainly influenced by surface properties. It can affect the absorption of apatite or proteins and also influence cell behavior by inducing a cascade of events leading to the enhancement of cell affinity, proliferation, and differentiation [3–6]. Therefore, the chemistry of the polymers, especially the surface properties, greatly influences cell attachment and biocompatibility. PLLA synthesized polymers possess a relatively hydrophobic surface, with a static water contact angle of approximately 80°, which results in its low cell affinity (especially for anchorage-dependent cells, like mesenchymal stromal cells) and limits its application as a tissue engineering scaffold [7]. To overcome such challenges, optimizing the surface chemistry of the material to improve the initial stage of cell adhesion is needed.

Modification of polymer surfaces can be achieved either by the design of copolymerization or binding of active molecules [5]. However, because of a lack of functional groups, PLLA cannot be easily modified with biologically active moieties [8]. Copolymerization seems to be a more reliable method. Because of its excellent biocompatibility and nonimmunogenicity, poly(ethylene glycol) (PEG), an important hydrophilic and water soluble polymer, has been widely applied in many biomedical fields, including tissue engineering, surface modification, and drug delivery [9]. Therefore, efforts can be made to modify PLLA using PEG, in order to make the hydrophobic surface more hydrophilic, and thus improving the affinity for proteins and cells.

Mechanisms underlying the cell adhesion to the biomaterial surfaces involve a series of related cellular signaling genes, which are regulated and then followed by a cascade of intracellular molecular events. Hence, the improvement of cell affinity through modification of biomaterial surfaces would likely be due to the change of the expression of adhesion-related genes. Based on these facts, a PEG/PLLA copolymer was prepared and cell behaviors were evaluated in the early cell attachment phase. In total, 84 cell adhesion molecular and extracellular matrix (ECM) genes were assayed to unveil the molecular mechanisms related to cell adhesion.

## 2. Materials and Methods

### 2.1. Preparation of PLLA and the MPEG-*b*-PLLA Copolymer Films

The MPEG-*b*-PLLA copolymer was prepared according to the procedures reported by Gotsche and coworkers [10]. Briefly, Diblock copolymer of MPEG-PLLA was prepared by the ROP of l-lactide in the presence of MPEG and stannous octoate (Sn(Oct)_2_). The –OH end-group of the diblock copolymer MPEG-PLLA-OH was then converted into –Phe-NBOC; finally the –Phe-NBOC group was treated by trifluoroacetic acid (TFA) to get the free aminoterminated MPEG-*b*-PLLA copolymer. The MPEG-*b*-PLLA copolymer film was prepared following the following procedures: commercial high molecular weight PLLA (*M*_*w*_ = 100,000–150,000 g/mol) and the diblock copolymers of MPEG-*b*-PLLA were dissolved in chloroform (5 wt.% of MPEG-*b*-PLLA being blended with 95% PLLA); the solution was then casted onto glass dishes (35 mm in diameter), allowing the solvent to evaporate completely in air. Pure PLLA films were prepared the same way and used as controls, while tissue culture polystyrene (TCPS) served as the positive control.

### 2.2. Isolation and Expansion of Human Bone Marrow Stromal Cells (hBMSCs)

hBMSCs were isolated and cultured based on protocols from previous studies [11, 12]. Briefly, bone marrow was obtained from patients (50–60 years old) undergoing hip and knee replacement surgery with informed consent given by all donors. The procedure was approved by the Ethics Committee of Queensland University of Technology. Lymphoprep was added to isolate the mononuclear cells from the bone marrow by density gradient centrifugation (Axis-Shield PoC AS, Oslo, Norway). The obtained cells were seeded into tissue culture flasks containing DMEM supplemented with 10% FBS and 1% penicillin/streptomycin and incubated at 37°C in a humidified CO_2_ incubator. The culture medium was changed every 3 days until the primary mesenchymal cells reached 80% confluence. The unattached hematopoietic cells were removed through medium change. The confluent cells were routinely subcultured by trypsinization. Only early passages (p3–5) of cells were used in this study.

### 2.3. Cell Morphology in Initial Adhesion

The morphology of hBMSCs in the early adhesion process was observed. hBMSCs were serum-starved for 4 h and then seeded on the polymer films and TCP surface. After 1 h, 2 h, and 24 h of incubation, the samples were washed 3 times with PBS and were then fixed firstly in 4% glutaraldehyde buffered in 0.1 M sodium cacodylate overnight, followed by 1% osmium tetroxide for 1 h. Dehydration was performed through a series of graded ethanol solutions and finally critical point dried in hexamethyldisilazane (HMDS). The samples were gold sputtered in a vacuum and then viewed with scanning electron microscope (SEM; FEI Company, Oregon, USA) at an accelerating voltage of 10 kV.

The total number of cells adhered on the films was quantified using the CyQuant NF Cell Proliferation Assay Kit (Invitrogen, Australia) following the manufacturer's instructions, and samples were detected using a fluorescence microplate reader (POLARstar Optima, Germany) with the excitation wavelength at 485 nm and emission wavelength at 585 nm.

To further evaluate the proliferation potential of the attached cell, cells were incubated for 3 d and then fixed in 4% paraformaldehyde for 15 min. Samples were washed 3 times in PBS and then dyed by crystal violet for 10 min and finally observed by light microscope (Carl Zeiss MicroImaging GmbH, Gottingen, Germany) at a magnification of 20x.

### 2.4. Cytoskeleton Organization

Actin fiber distribution in hBMSCs attached on the polymer films and TCP surface was observed. After serum starving for 4 h, the cells were seeded and cultured for 1 and 24 h on the surfaces. PBS was applied to remove the unattached cells. The remaining attached cells were fixed with 4% paraformaldehyde for 20 min and rinsed with PBS. The cells were permeabilized in PBS containing 0.2% Tween 20 for 20 min and then incubated with 0.1 mg/mL propidium iodide (Sigma-Aldrich, Missouri, USA) for 5 min to stain the cell nuclei. After being thoroughly washed with PBS, the cells were incubated in Phalloidin Alexa Fluor 488 (Life technologies, California, USA) for 30 min to stain filamentous actin, washed 3 times with PBS, and mounted in 10 mL of mounting solution (Life technologies, California, USA). Images were captured with a confocal laser scanning microscope (Leica, Wetzlar, Germany).

### 2.5. Extracellular Matrix and Adhesion Gene Expression Analysis by RT-qPCR

Quantitative RT-PCR was carried out using an RT^2^ Profiler plate (Biocompare, California, USA). The plate contained a set of primers for 84 genes of extracellular matrix and adhesion molecules. Briefly, hBMSCs were seeded on the films of MPEG-*b*-PLLA, PLLA, and TCPS surfaces. After 24 h of incubation, the total RNA sample was isolated using Trizol Reagent (Life technologies, California, USA) according to the manufacturer's instructions. The cDNA samples were prepared from the isolated RNA using the reverse transcription first strand kit (Finnzymes, Thermo Scientific, Massachusetts, USA) and then added to the RT^2^ quantitative PCR master mix containing SYBR Green and a reference dye. The above mixture was then aliquoted across the PCR array templates, which contained 84 pathway-specific genes plus housekeeping genes. The RT-PCR analysis was carried out using a thermal cycler (7300 ABI Prism, Applied Biosystems, California, USA). Each sample was analyzed in triplicate. Relative gene expression values were analyzed using the SuperArray web-based software package (available at http://www.superarray.com/). Gene expressions that were increased or decreased with a *P* value less than 0.05 and at least a 2-fold difference were considered to have significant differential expression.

### 2.6. Statistical Analysis

Original data were collected and normalized according to the cell numbers, cell percentages, and fluorescence values. A single factor analysis of variance (ANOVA) technique was used to assess the statistical significance of the results. Scheffe's method was employed for multiple comparison tests. The level of significance was set at *P* < 0.05.

## 3. Results

### 3.1. Cell Attachment and Spreading

**Figure 1:**
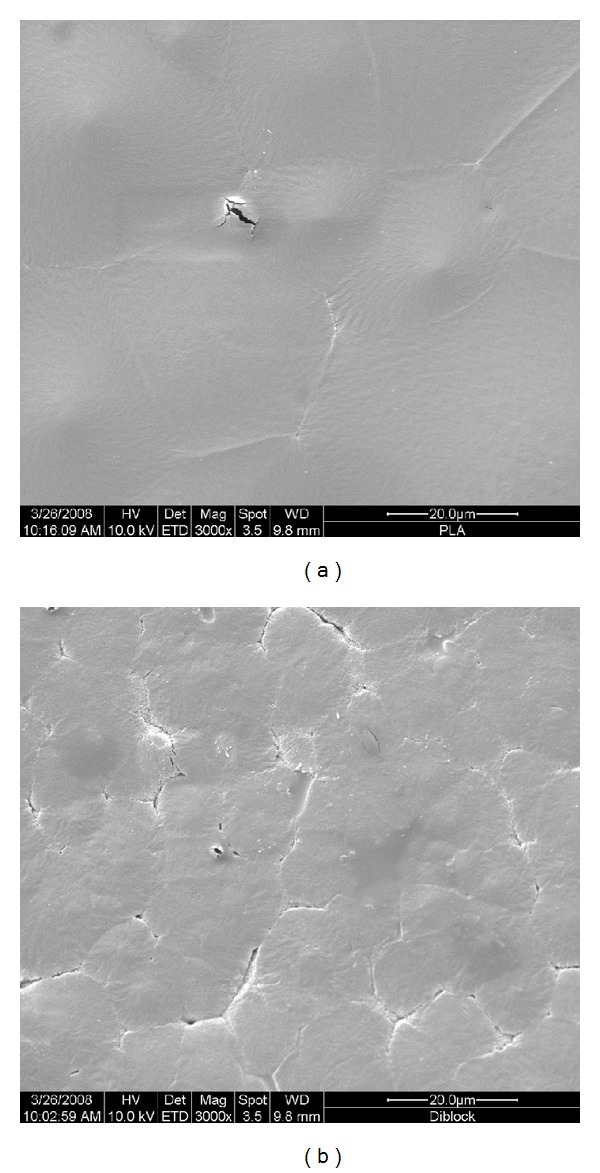
Surface morphology of the PLLA (a) and MPEG modified PLLA (b).

**Figure 2:**
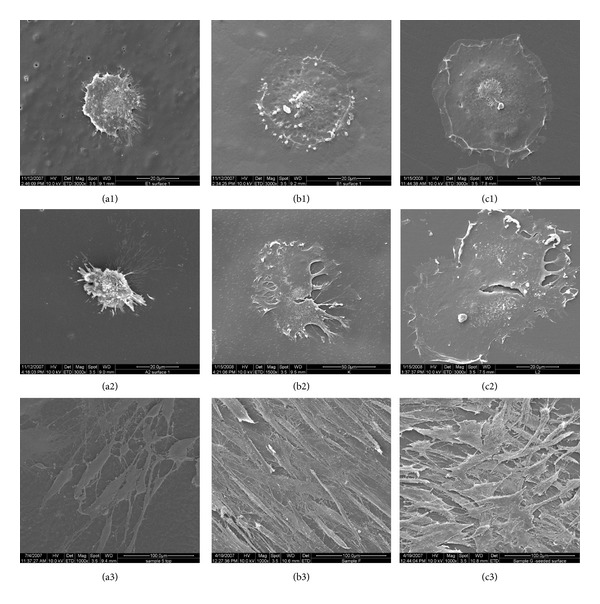
Attachment of hBMSCs on the surface of PLLA (a1–a3), MPEG-*b*-PLLA (b1–b3), and TCPS (c1–c3) after 1 h (a1, b1, and c1), 2 h (a2, b2, and c2), and 24 h (a3, b3, and c3).

Cell attachment and spreading are crucial for cell growth and differentiation and therefore become vital factors when evaluating the biocompatibility of a biomaterial. As shown in Figure 1, the surface of PLLA was smoother than that of MEPG-*b*-PLLA, which revealed a more significant groove and ridge morphology. After 1 h incubation of hBMSCs on the polymer surfaces, the cells were evenly spread and had a flatted appearance on the diblock membrane (Figure 2 (b1)), similar to the cell morphology on the TCP surface (Figure 2 (c1)). However, cells on PLLA (Figure 2 (a1)) appeared to be less spread out and had less regular cell morphology.

After 2 h incubation, the cell morphology on the diblock film became flatter and had a greater number of cytoplasmic extensions over the entire polymer surface compared to 1 h incubation (Figure 2 (b2)). On the contrary, cells on the PLLA surface appeared spheroid with filopodia and were less spread compared with those subjected to the 1 h incubation (Figure 2 (a2)). All 3 surfaces had more cells attached after another 24 h incubation; however, more cells were observed on the diblock film surface (Figure 2 (b3)) compared with PLLA (Figure 2 (a3)), while the number of attached cells on the diblock film surface was similar to that of the TCP surface (Figure 2 (c3)). The cells grew well on both diblock and TCP surfaces. After 3 d expansion, both surfaces reached at least 90% confluence (Figures 4(b) and 4(c)); in contrast, the PLLA film was less beneficial for the proliferation of hBMSCs, with less than 50% confluence (Figure 4(a)).

**Figure 3:**
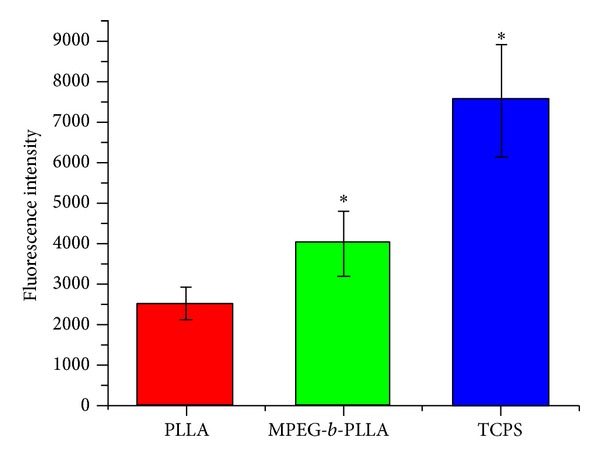
Quantitative analysis of hBMSCs attachment on the surface of PLLA, MPEG-*b*-PLLA, and TCPS after 1 h using CyQuant NF Cell Proliferation Assay Kit, *Significant difference (*P* < 0.05).

**Figure 4:**
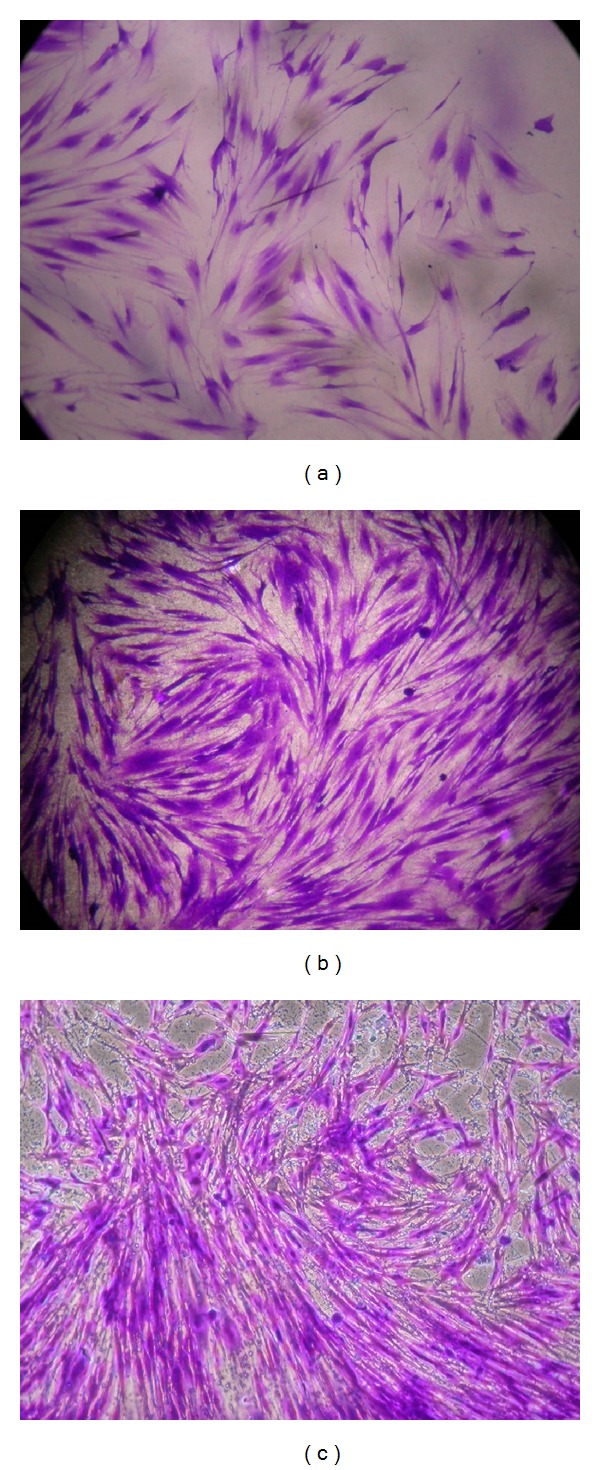
Proliferation of hBMSCs on the surface of PLLA (a), MPEG-*b*-PLLA (b), and TCPS (c) after 3 d.

Quantitative analysis of hBMSCs attachment onthe surface of PLLA, MPEG-*b*-PLLA, and TCPS after 1 h showed similar trend. MEPG-*b*-PLLA group showed higher fluorescence intensity than the PLLA group. However, when compared with the TCP group, the fluorescence intensity of MPEG-*b*-PLLA group was smaller (Figure 3).

**Figure 5:**
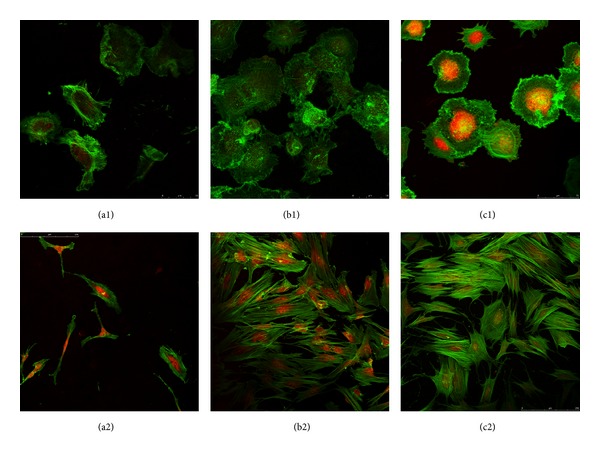
Representative confocal laser scanning microscopy images of hBMSCs cultured on polymer films and TCPS after 1 h and 24 h. Actin is shown in green and nuclei are shown in red. (a1–c1) separately represents PLLA, MPEG-*b*-PLLA, and TCPS for 1 h, (a2–c2) for 24 h.

The cellular expression of F-actin fibers on the different surfaces is shown in Figure 5. In the first hour, cells cultured on the surface of MPEG-*b*-PLLA diblock copolymer film (Figure 5 (b1)) and TCPS surfaces (Figure 5 (c1)) had a spreading shape, with the formation of pseudopods. The F-actin fibers were abundant and well organized and were arranged in a linear orientation in the cells (Figure 5 (b1)). On the contrary, most cells on the PLLA surface (Figure 5 (a1)) had a more spheroid morphology with smaller size and fewer pseudopods compared with cells on the other surfaces; F-actin was observed, but the distribution was random and disordered (Figure 5 (a1)). After 24 h incubation, the cells on the diblock copolymer and TCP surfaces had a well-extended structure (Figures 5 (b2) and 5 (c2)). Abundant actin filaments were clearly visible passing across the cytoplasm and over the nucleus, and individual actin bundles were easily distinguishable. In contrast, the cells on the PLLA polymer film shrunk to a smaller size, and the F-actin filaments appeared as ring-shaped dots that were sparsely distributed around the cell boundary or nuclei (Figure 5 (a2)).

### 3.2. Extracellular Matrix and Adhesion Gene Expression in Initial Attached hBMSCs

**Figure 6:**
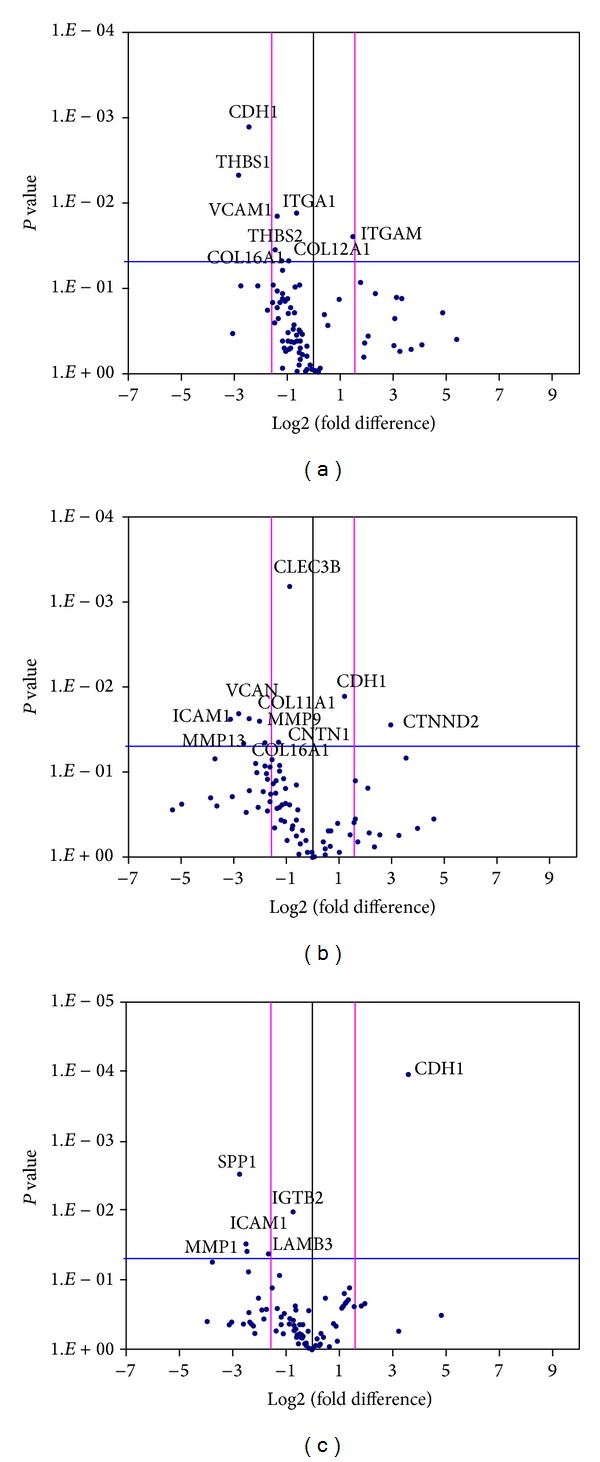
Extracellular matrix and adhesion by attached hBMSCs: PLLA/TCPS (a), MPEG-*b*-PLLA/TCPS (b), and MPEG-*b*-PLLA/PLLA (c). The plots indicate a fold-change in gene expression displaying the genes which changed significantly (*P* < 0.05) (above the blue line) and which changed by more than 3-fold (beyond the pink line).

Compared with the TCP surface, both MPEG-*b*-PLLA and PLLA surfaces showed a downregulation of most adhesion genes (Table 1 and Figure 6). On the MPEG-*b*-PLLA surface, 7 out of 9 significantly regulated genes were downregulated, including CLEC3B, COL11A1, COL16A1, ICAM1, MMP9, MMP13, and VCNA. The upregulated genes were CDH1 and CTNND2, two key components for the cadherin-catenin complex. Similar gene profiles were observed in cells on the PLLA surface, with 7 out of 8 genes downregulated, including CDH1, THBS1, VCAM1, THBS2, ITGA1, Col16A1, and Col12A1. ITGAM was the only gene upregulated in these cells. When compared the PLLA and MPEG-*b*-PLLA surfaces, CDH1 was significantly upregulated in hBMSCs attached on the MPEG-*b*-PLLA copolymer surfaces with a fold change of up to 12. Five genes were downregulated, including ICAM1, ITGB2, LAMB3, MMP1, and SPP1.

## 4. Discussion

The attachment of mesenchymal stem cells to biomaterials is the primary step of the cascade of events leading to new bone formation. To improve the affinity of PLLA, MPEG was used to modify the PLLA surface in this study, in order to achieve a more hydrophilic surface. The modified polymer surface demonstrated an increased number of attached cells and enhanced cell spreading at the early phase and further improved cell proliferation. It was observed that a modified polymer surface could directly influence the ECM and adhesion gene expression of hBMSCs and consequently enhance cell affinity.

Initial cell attachment is mainly mediated by the weak and unstable physicochemical connection, which is greatly influenced by the surface morphology (roughness) [13], surface energy, surface charge [14], and hydrophilicity/hydrophobicity [15]. MPEG modification changed all these parameters of PLLA, which offered a more attachment-friendly environment for hBMSCs. The modified PLLA surface became rougher, providing suitable groves for the cell settling and accumulation of fibronectin, and therefore improved cell attachment [16, 17]. The incorporation of MPEG can also produce well-solvated polymer brushes on the surface at biomaterial-aqueous solution layers; this offers more degrees of freedom for cell adhesion molecules by working as a spacer [18, 19]. Cells tend to attach to the surface with higher wettability, likely owing to the preference of fibronectin to the hydrophilic surface [20]. Through copolymerizing a hydrophobic polymer, PLLA, with a hydrophilic polymer, MPEG, the copolymer surface became more hydrophilic and created some areas that were both hydrophobic and hydrophilic, which would be beneficial for the absorption and attachment of adhesion proteins with both hydrophobic and hydrophilic functional domains.

After the initial unstable attachment on the surface of materials, cells further secret adhesion-related proteins (extracellular matrix, cell membrane, and cytoskeleton proteins) and form cell junctions, to build up a stable and strong attachment (cell-substrate adhesion) with the materials. Cell morphology will be accordingly changed, from a small rounded shape, followed by a larger flattened appearance, to the natural stretched form. In this study, cells on diblock copolymer surfaces exhibited a more spread-out morphology than those on the PLLA surface. A well-organized actin cytoskeleton was observed in hBMSCs on the diblock copolymer and TCPS surfaces compared with cells on the PLLA copolymer surface. These results indicate that diblock polymer surfaces elicited some effects on the actin cytoskeleton, providing more favorable conditions for the formation of cell-substrate adhesion and spreading of hBMSCs. Actin is also known to be involved in other activities, such as cytoplasmic streaming, cell division, cell surface events, and motility [21]. The actin cytoskeleton plays important roles in the enhancement of cell affinity by the diblock copolymer.

In addition, the formation of attachment structures can also initiate several key events, which affect cell signaling, nuclear organization, and cytoskeletal formation [22]. For example, the binding of ligands to the integrin receptors could activate the MAPK signaling pathway [23], upregulating related gene expressions for cell proliferation. Therefore, understanding the effect of modified polymer surfaces on initial adhesion gene expression will eventually reveal the mechanisms responsible for controlling the subsequent behavior of hBMSCs. With these in mind, we further analyzed 84 ECM and adhesion molecule genes, to unveil the molecular mechanisms of modified PLLA surfaces on cell adhesion. In this study, the most significantly influenced (upregulated) cell adhesion gene in the MPEG-*b*-PLLA modified PLLA compared with the PLLA surface was CDH1 (12.14-fold). Compared with the TCPS surface, CTNND2 was the most significantly influenced (upregulated) cell adhesion gene in the modified PLLA (8.6-fold).

Cadherins are transmembrane proteins with a five-repeat extracellular domain, a transmembrane region, and a conserved cytoplasmic domain; Ca^2+^ is required for cadherin protein conformation and is thought to involve multiple cis-dimers of cadherin that form transoligomers between cadherins on adjacent surfaces [24]. However, binding between cadherin extracellular domains is weak, and strong adhesion requires the formation of adherens junctions. The transmembrane core of adherens junctions is composed of 1-cadherin, whose cytoplasmic domain associates with catenins, which coordinate actin dynamics to assemble and stabilize these junctions [25]. CDH1 is known to encode 1-cadherin (also known as E-cadherin), while CTNND2 encodes delta-2-catenin. The upregulation of both genes together with the well-organized actin cytoskeleton in the diblock copolymer indicated that the enhancement of adhesion is likely related to the formation of adherens junctions. As the adherens junctions are assembled, the cadherin-catenin complex could also actively transform the actin cytoskeleton [26], resulting in good protrusion and stretching of hBMSCs and ultimately expanding the cell attachment on the modified polymer surface.

The cadherin-catenin complex was also found to be responsible for the recruitment and activation of some signaling molecules. The Wnt signaling pathway is one typical example [27, 28]. Delta-catenin is required for the Wnt pathway signaling [27, 28]. It enhances the binding of Kaiso transcription factor to the repressed promoters, relieving the inhibition of the *β*-catenin-Tcf-4 transcriptional complex [27]. The depletion of delta-catenin inhibits axin recruitment, affects LRP5/6 phosphorylation, and abolishes CK1*ε* binding to LRP5/6 and CK1*ε* activation upon Wnt3a stimulation [28]. 1-cadherin is associated with Wnt coreceptor LRP5/6 and can bind to beta-catenin (core component for the canonical Wnt signaling pathway) [29, 30]. The activated CK1*ε* can phosphorylate 1-cadherin, resulting in the release of beta-catenin, activating the canonical Wnt signaling pathway [29, 30]. This means that the high expression of both genes might activate the Wnt signaling pathway, which could then enhance the proliferation and osteogenic differentiation of hBMSCs on the modified polymer surface.

## 5. Conclusions

MPEG-*b*-PLLA copolymers could upregulate the adhesion molecule complex cadherin-catenin genes, resulting in improving the initial BMSC attachment and spreading via the well-organized actin cytoskeleton in initial attachment of hBMSCs.

## Figures and Tables

**Table 1:** Differential expression of extracellular matrix and adhesion genes by attached hBMSCs among PLLA, MPEG-*b*-PLLA, and TCP surfaces.

Gene	MPEG-PLLA/PLLA		MPEG-PLLA/TCP		PLLA/TCP	
	Fold change	*t*-test	Fold change	*t*-test	Fold change	*t*-test
		*P* value		*P* value		*P* value
ACTB					−2.98	0.0468
CDH1	12.14	0.0001	2.25	0.0131	−5.39	0.0022
CLEC3B			−1.58	0.0007	−1.64	0.0455
COL11A1			−5.38	0.0236		
COL16A1			−3.57	0.0454		
CTNND2			7.84	0.0281		
ICAM1	−5.56	0.031	−8.58	0.0241		
ITGAM					2.73	0.0173
ITGB2	−1.65	0.0106				
LAMB3	−3.12	0.0423				
MMP1	−5.54	0.0389				
MMP9			−3.99	0.0253		
MMP13			−6.26	0.0459		
SPG7					−1.45	0.0244
SPP1	−6.62	0.003				
THBS1					−7.06	0.0040
THBS2					−2.70	0.0273
VCAM1					−2.63	0.0101
VCNA			−7.04	0.0205		
